# Supplementary material for: Galectin-3 is required for the microglia-mediated brain inflammation in a model of Huntington’s disease
Source: Nat Commun. 2019 Aug 2;10:3473. doi: 10.1038/s41467-019-11441-0 (PMC6677843; doi:10.1038/s41467-019-11441-0)
Supplement: Supplementary file 1 — Supplementary Information [file 41467_2019_11441_MOESM1_ESM.pdf]

Supplementary Information

Galectin-3 is required for the microglia-mediated brain inflammation in Huntington's disease

Siew et al.

Supplementary Fig. 1 (Related to Fig. 1)

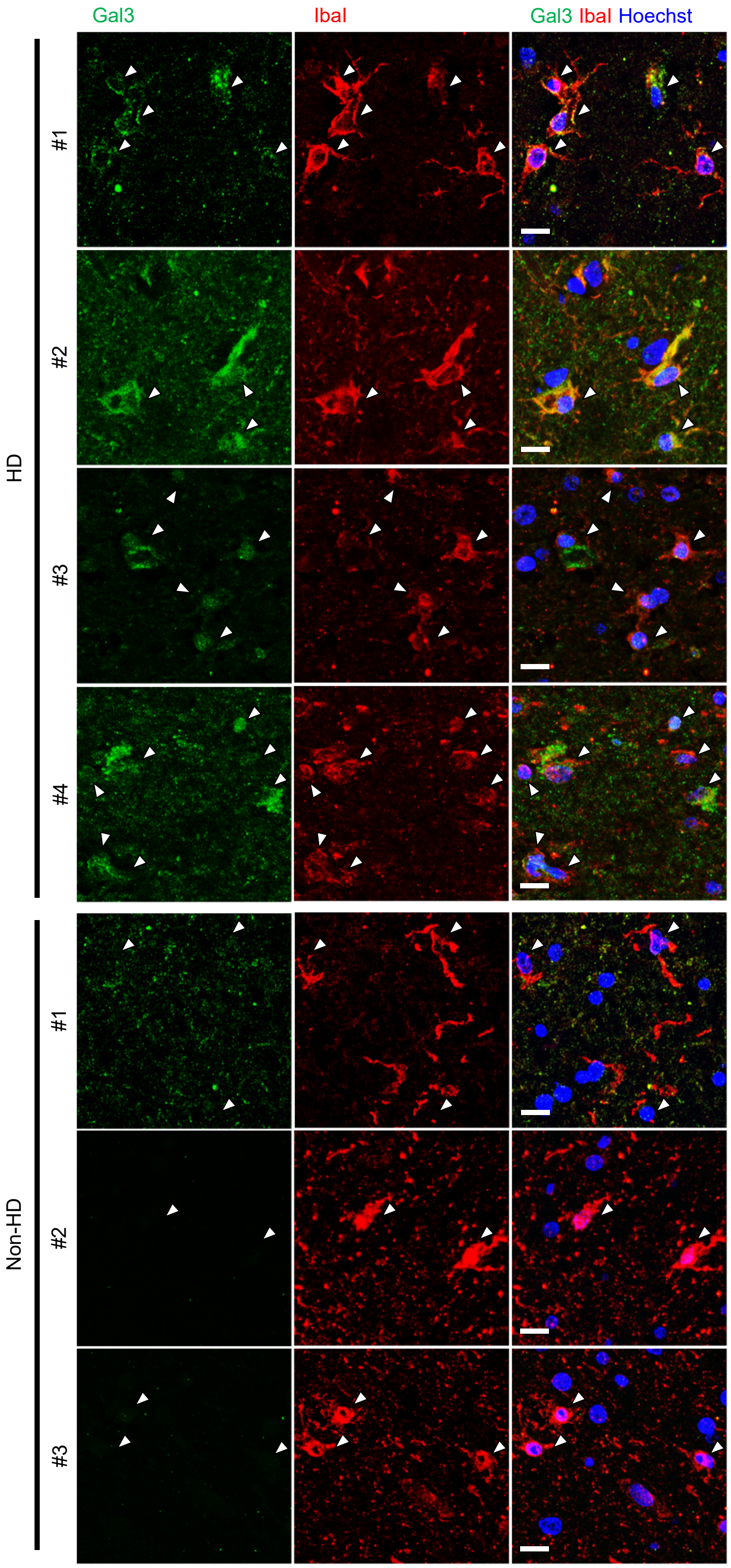

Supplementary Figure 1. Expression of Gal3 on microglia in the brain of HD patients. Brain sections (caudate putamen) from non-HD and HD patients were stained with anti-Gal3 antibody (green) and a microglial marker (IbaI, red). Nuclei were stained with Hoechst (blue). Arrows mark the IbaI-positive cells (i.e., microglia). Scale bar: 10 µm.

Supplementary Fig. 2
(Related to Fig. 2)

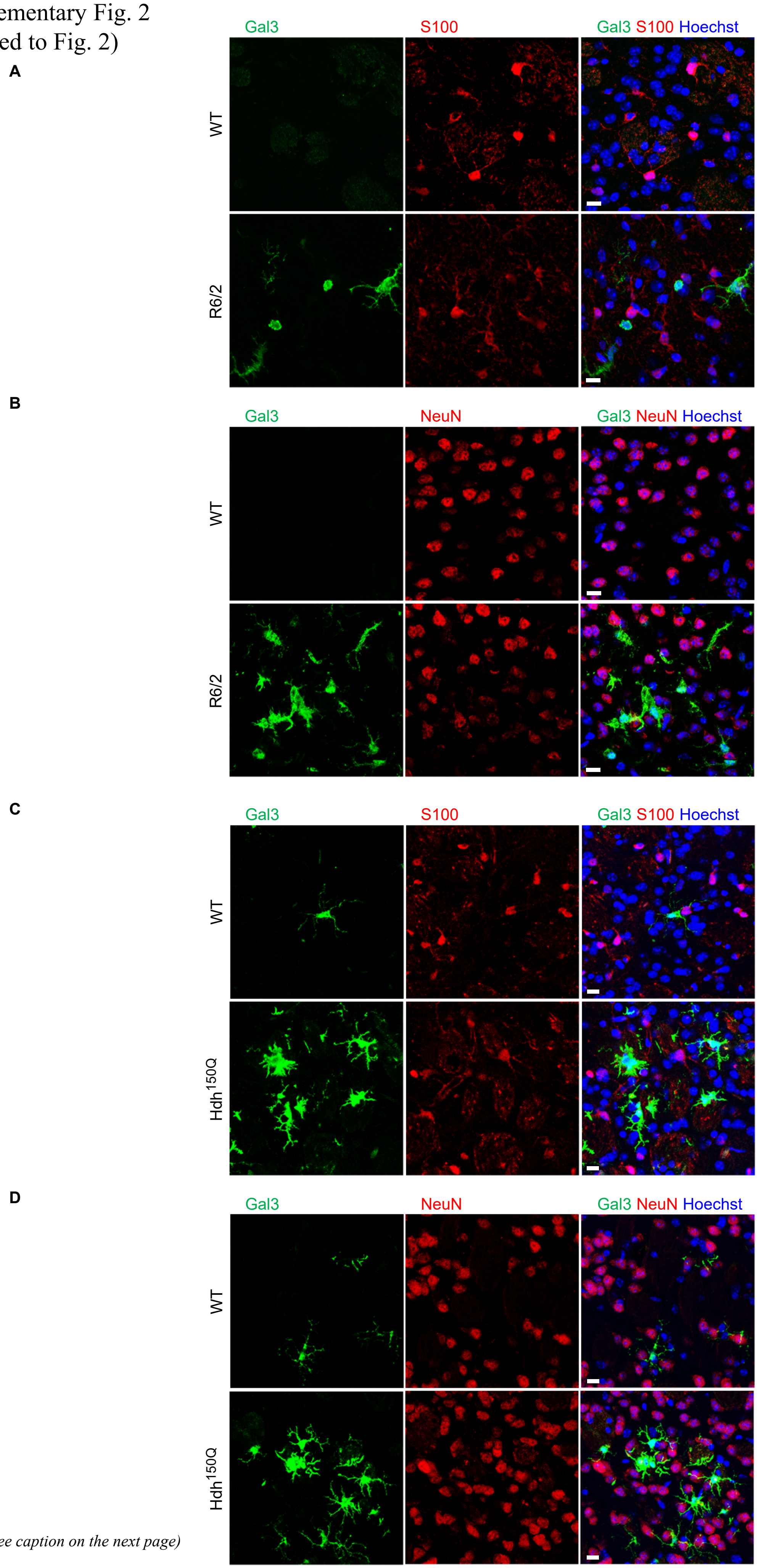

(see caption on the next page)

Supplementary Figure 2. *(see image on the previous page)*

Expression of Gal3 in the striatum of R6/2. Brain sections from WT and R6/2 mice were stained with an anti-Gal3 antibody (green) and **(A)** an astrocyte marker (S100, red) or **(B)** a neuronal marker (NeuN, red). Brain sections from WT and Hdh^{150Q} mice were stained with an anti-Gal3 antibody (green) and **(C)** an astrocyte marker (S100, red) or **(D)** a neuronal marker (NeuN, red). Nuclei were stained with Hoechst (blue). Scale bar: 10 µm.

Supplementary Fig. 3 (Related to Fig. 2)

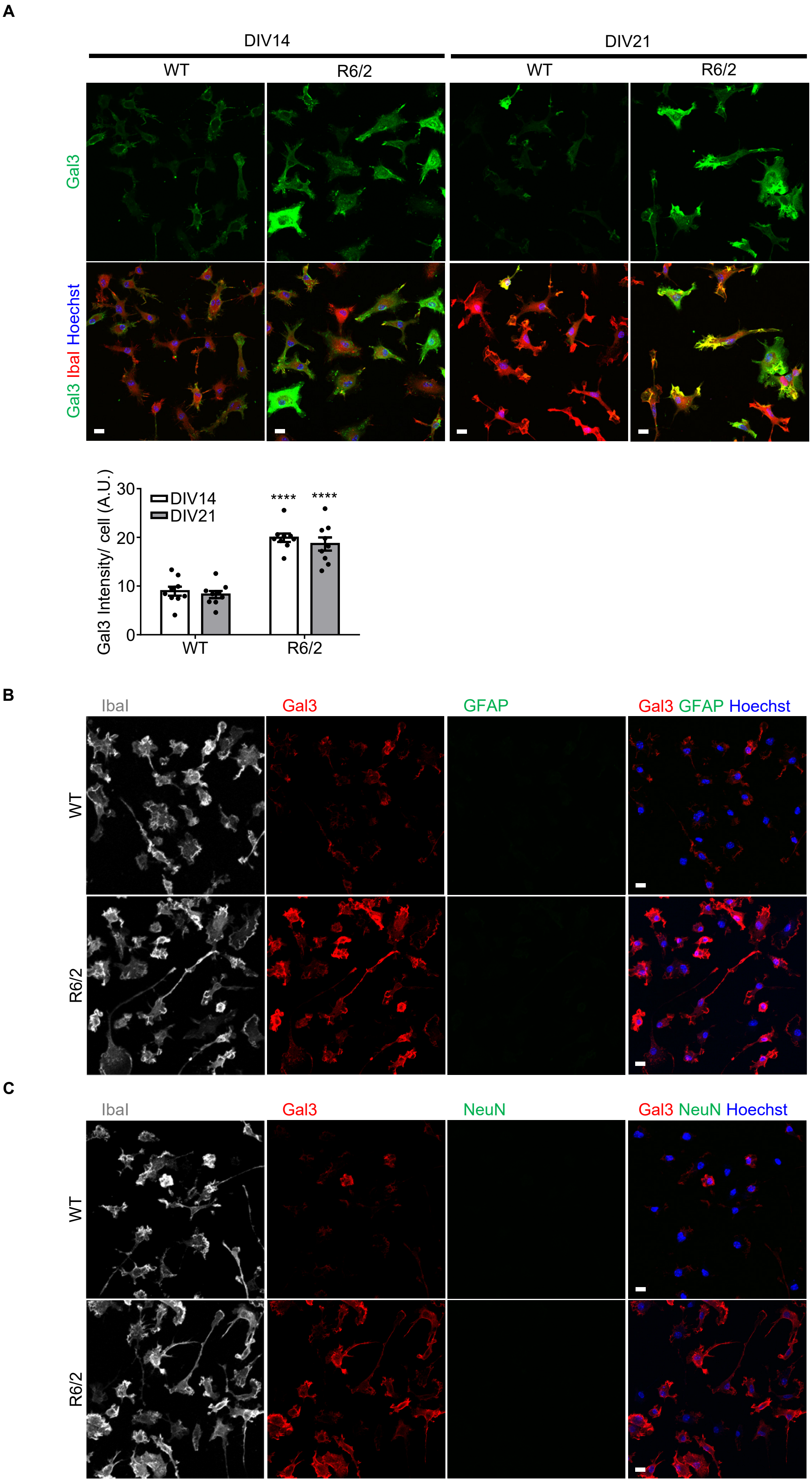

(see caption on the next page)

Supplementary Figure 3. *(see image on the previous page)*
Comparative study of Gal3 on primary microglia at DIV14 and DIV21. (A) Primary microglia from R6/2 and their littermate controls (WT) were harvested from mix glial culture on 14 and 21 DIV. The isolated cells were cultured for 48 hours and were fixed for immunofluorescence staining of Gal3 (green) and a microglial marker (red). Nuclei were stained with Hoechst (blue). Primary microglia cultures from R6/2 mice and littermates were stained for Gal3 (red), IbaI (gray) and **(B)** an astrocyte marker (GFAP, green) or **(C)** a neuronal marker (NeuN, green). Nuclei were stained with Hoechst (blue). Results were analyzed by two-way ANOVA followed by Tukey's post hoc test. Data are presented as the means ± SEM. *, Specific comparison between WT and R6/2 cells of the same time point; #, Specific comparison between cells from the same genotype but different time point; ****$P < 0.0001$. Same *P*-value denotation for #. Scale bar: 10 µm.

Supplementary Fig. 4 (Related to Fig. 3)

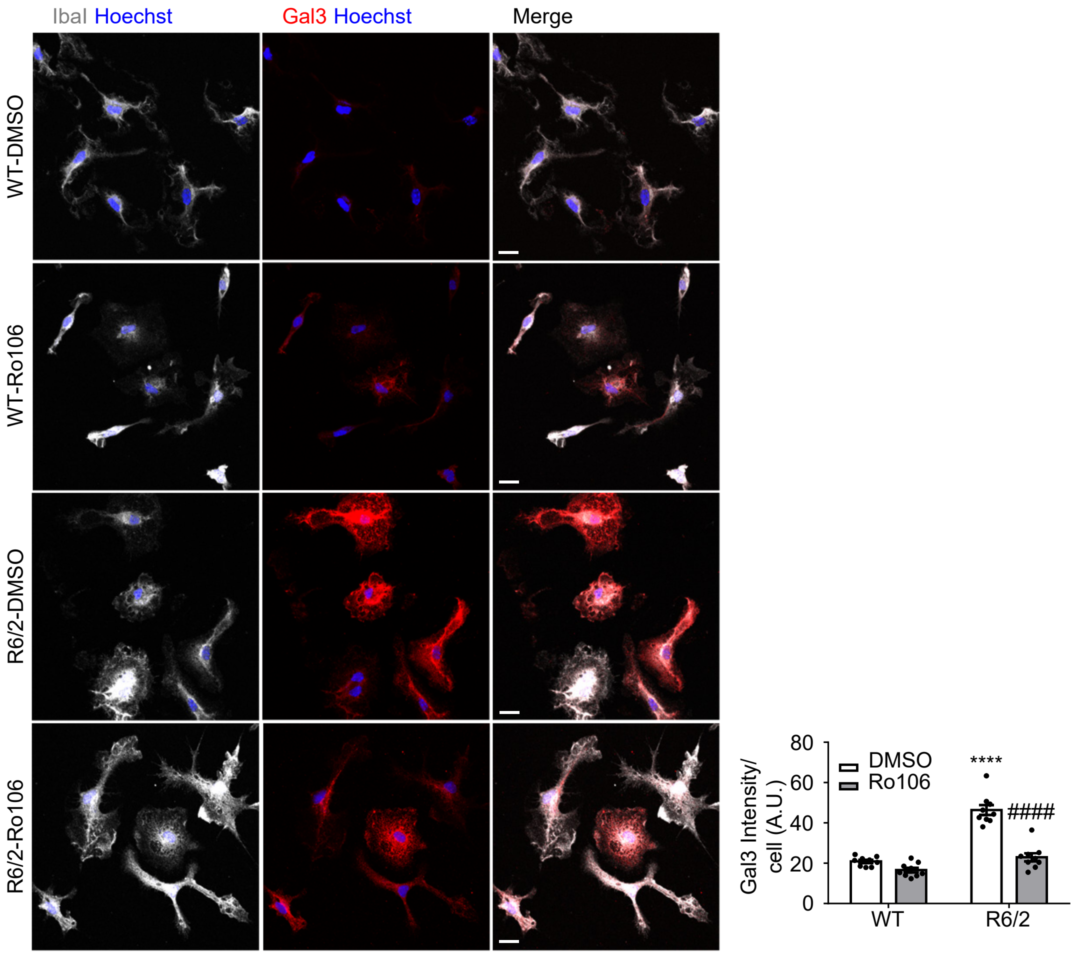

Supplementary Figure 4. Inhibition of the NFκB pathway by Ro106 reduces the abnormally up-regulated expression of Gal3 in primary R6/2 microglia. Primary microglia harvested from R6/2 and their littermate controls (WT) were treated with Ro 106-9920 (Ro106, 1 μM) or vehicle (0.1 % DMSO) as indicated for 24 hours (*n* = 9 in each group), and were fixed for immunofluorescence staining of Gal3 (red) and a microglial marker (gray). Nuclei were stained with Hoechst (blue). Results were analyzed by two-way ANOVA followed by Tukey's post hoc test. Data are presented as the means ± SEM. *, Specific comparison between WT and R6/2 cells of the same treatments; #, Specific comparison between the Ro106- and DMSO-treated groups of the same genotype; ****$P < 0.0001$. Same *P*-value denotation for #. Scale bar: 10 μm.

Supplementary Fig. 5 (Related to Fig. 3 & 4)

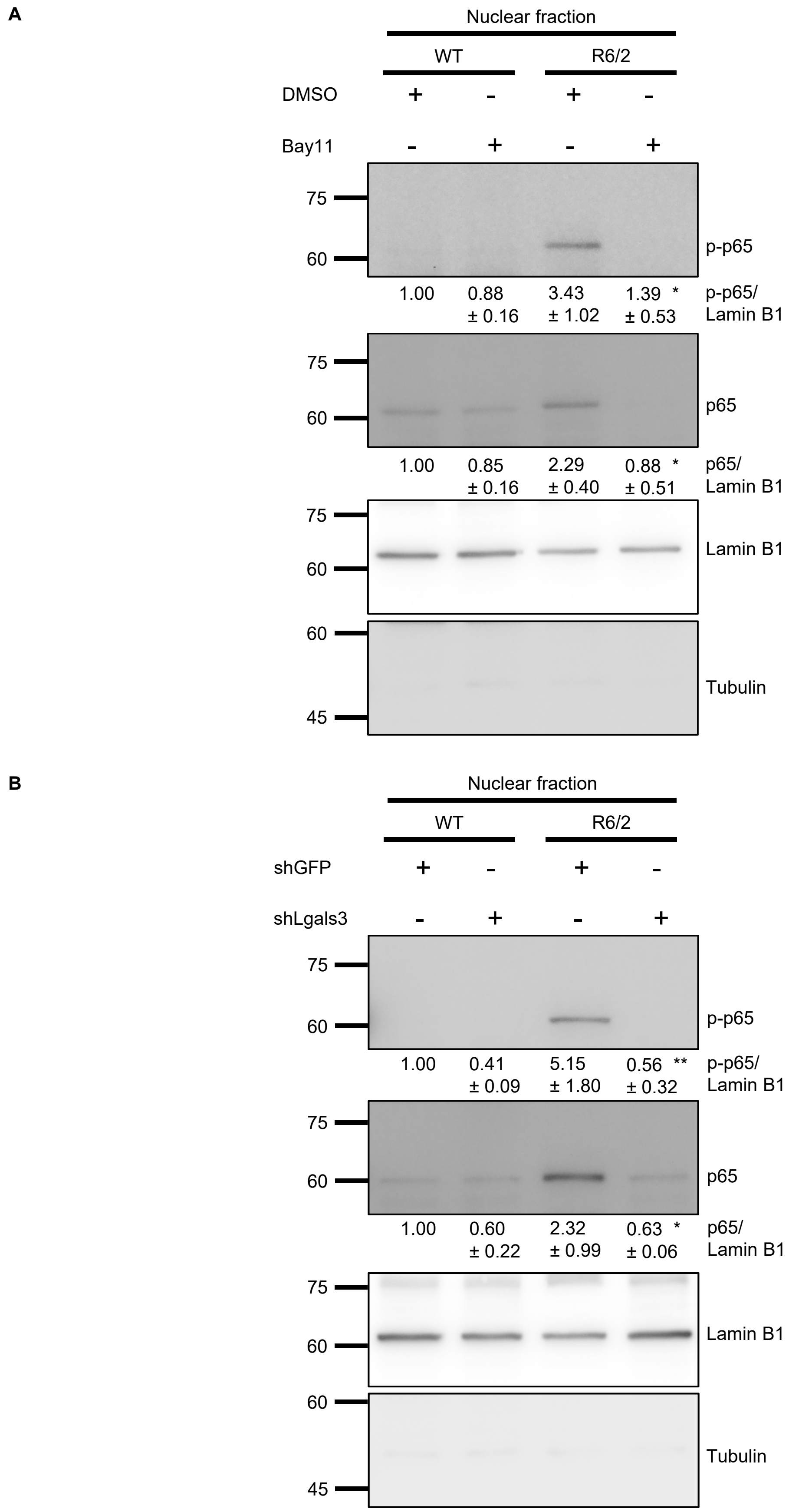

Supplementary Figure 5. Primary R6/2 microglia exhibited more NFκB activation than WT microglia. The levels of NFκB-p65 in the nucleus were analyzed by cellular fractionation and western blot. **(A)** Primary microglia harvested from R6/2 mice and their littermate controls (WT) were treated with Bay11-702 (3 μM) or vehicle (0.1% DMSO) as indicated for 24 hours (n =3). **(B)** Primary microglia were infected with the indicated lentiviruses expressing shRNA against Gal3 (i.e., sh*Lgals3*) to knockdown Gal3 or the control lentivirus (sh*GFP*), as described in the Methods section (n =3). Results were analyzed by two-way ANOVA followed by uncorrected Fisher's least significant difference test and presented as the means ± SEM, *, Specific comparison between the Bay11- and DMSO-treated groups or sh*GFP*- and sh*Lgals3*-treated groups of the same genotype; *$P < 0.05$, **$P < 0.01$.

Supplementary Fig. 6 (Related to Fig. 4 & 5)

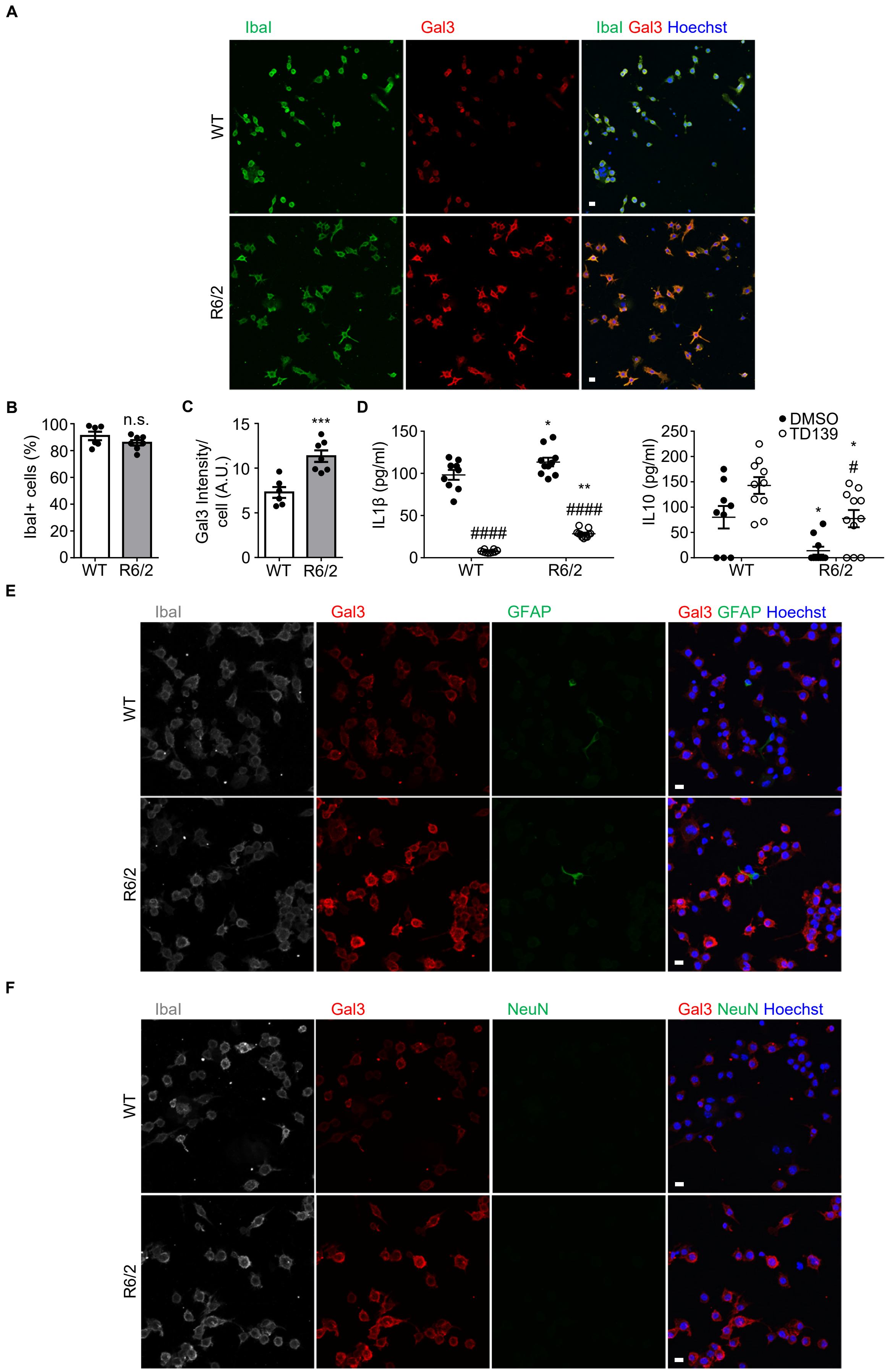

(see caption on the next page)

Supplementary Figure 6. *(see image on the previous page)*

Suppression of Gal3 at diseased stage/adult primary microglia. (A) Adult primary cultures of microglia were prepared from R6/2 and littermates as described in the Methods section and were stained with anti-Gal3 antibody (red) and anti-IbaI antibody (green). **(B, C)** The purity of isolated microglia and the level of Gal3 were determined by immunocytochemistry of IbaI-positive cells. **(D)** Adult primary microglia were treated with TD139 (20 μM) for 24 hours, and the supernatants were collected for ELISA (n = 9-11). Adult primary microglia cultures from R6/2 mice and littermates were stained for Gal3 (red), IbaI (gray) and **(E)** an astrocyte marker (S100, green) or **(F)** a neuronal marker (NeuN, green). Nuclei were stained with Hoechst (blue). Data in B & C are presented as the means ± SEM and analyzed by the unpaired Student's *t*-test. The results of D were analyzed by two-way ANOVA followed by Tukey's post hoc test. *, Specific comparison between WT and R6/2 cells of the same treatment; #, Specific comparison between the DMSO- and TD139-treated groups of the same genotype; *$P < 0.05$, **$P < 0.01$, ***$P < 0.001$, ****$P < 0.0001$. Same *P*-value denotation for #. n.s., not significant. Scale bar: 10 μm.

Supplementary Fig. 7 (Related to Fig. 6)

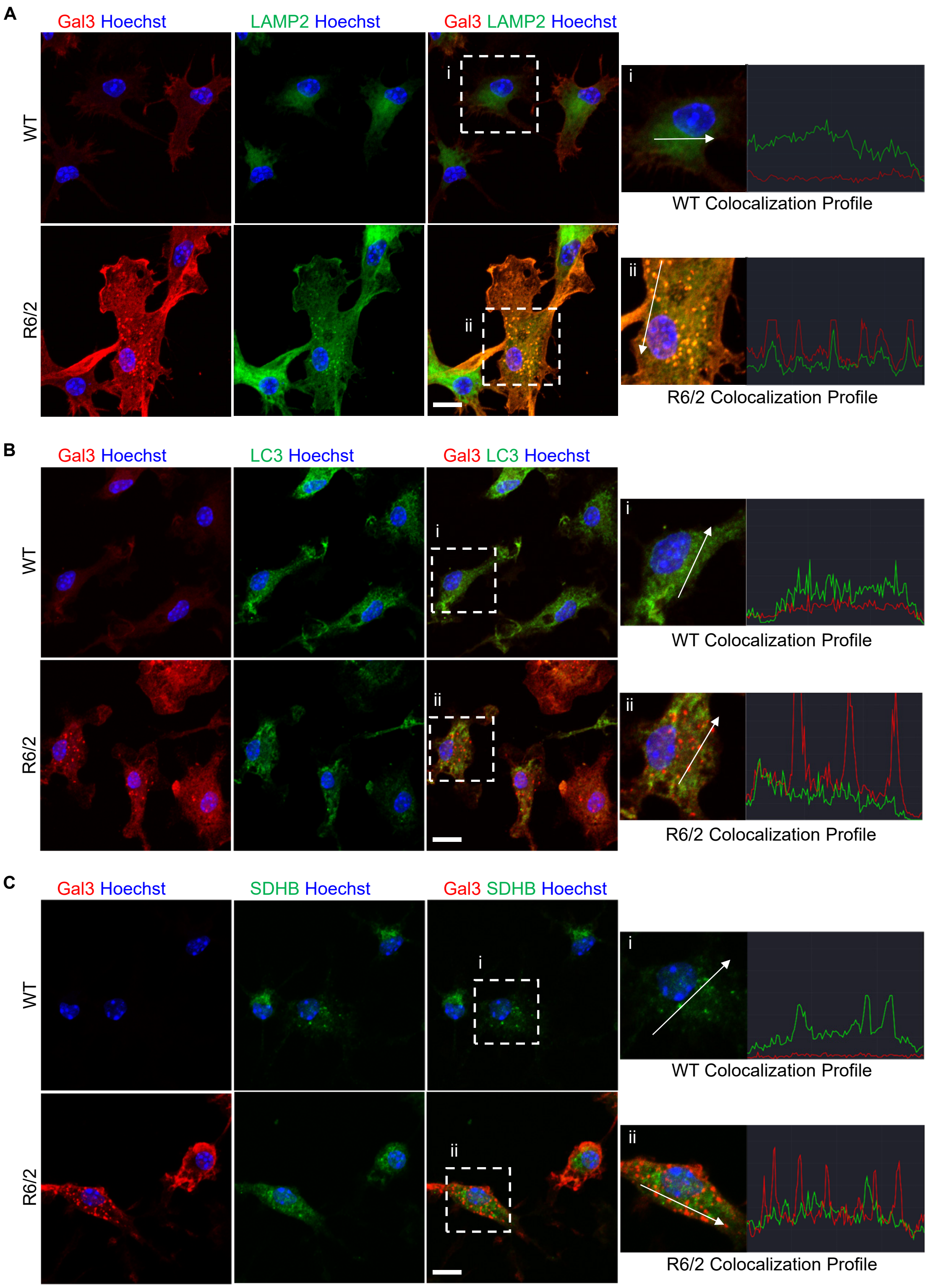

Supplementary Figure 7. Gal3 puncta were colocalized with LAMP2 (a lysosomal marker) but not with LC3 (an autophagosome marker) and SDHB (a mitochondrial marker) in R6/2 microglia. Immunofluorescence staining of Gal3 (red) with **(A)** LAMP2 (green) **(B)** LC3 (green), **(C)** SDHB (green) in primary microglia. The colocalization trace profiles of the puncta structures along the white straight lines were analyzed using Zen 2012 software. Scale bar: 10 µm.

Supplementary Fig. 8 (Related to Fig. 6)

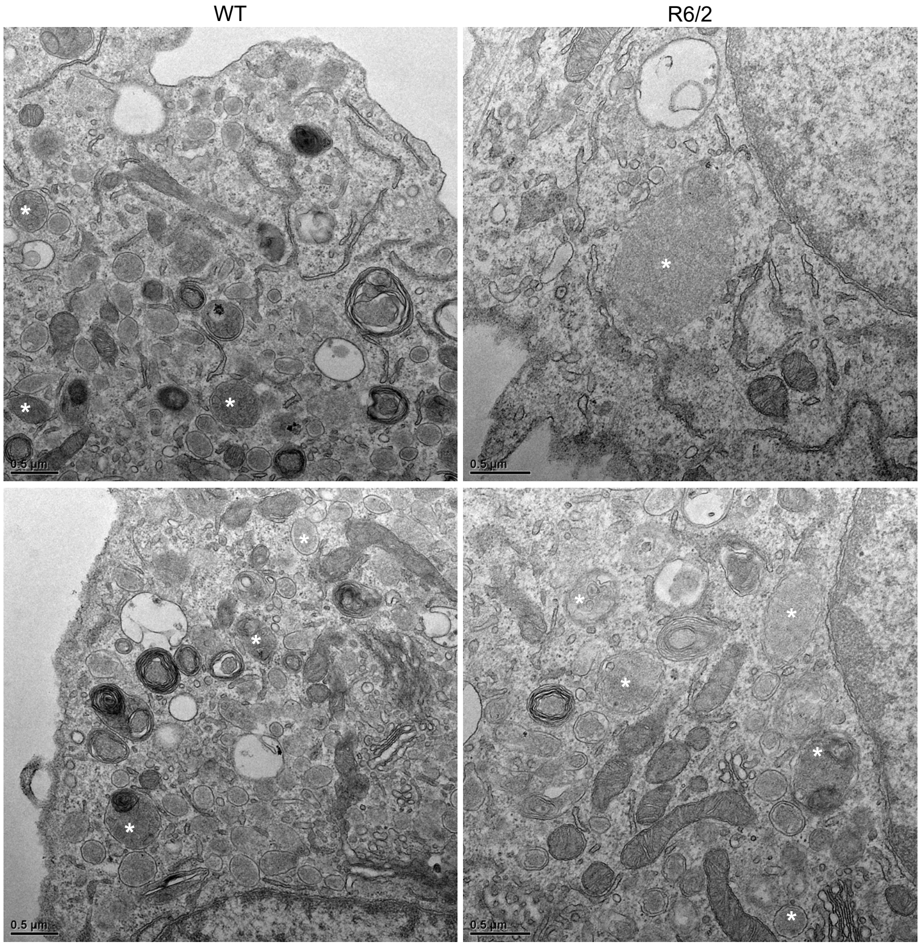

Supplementary Figure 8. Abnormal lysosomes in R6/2 microglia. Transmission electron microscopy of primary microglia isolated from R6/2 and littermates. Lysosomes-like structures in R6/2 microglia exhibited enlarged and irregular shape as compared to WT cells (marked by asterisk,*). Scale bar: 0.5 µm.

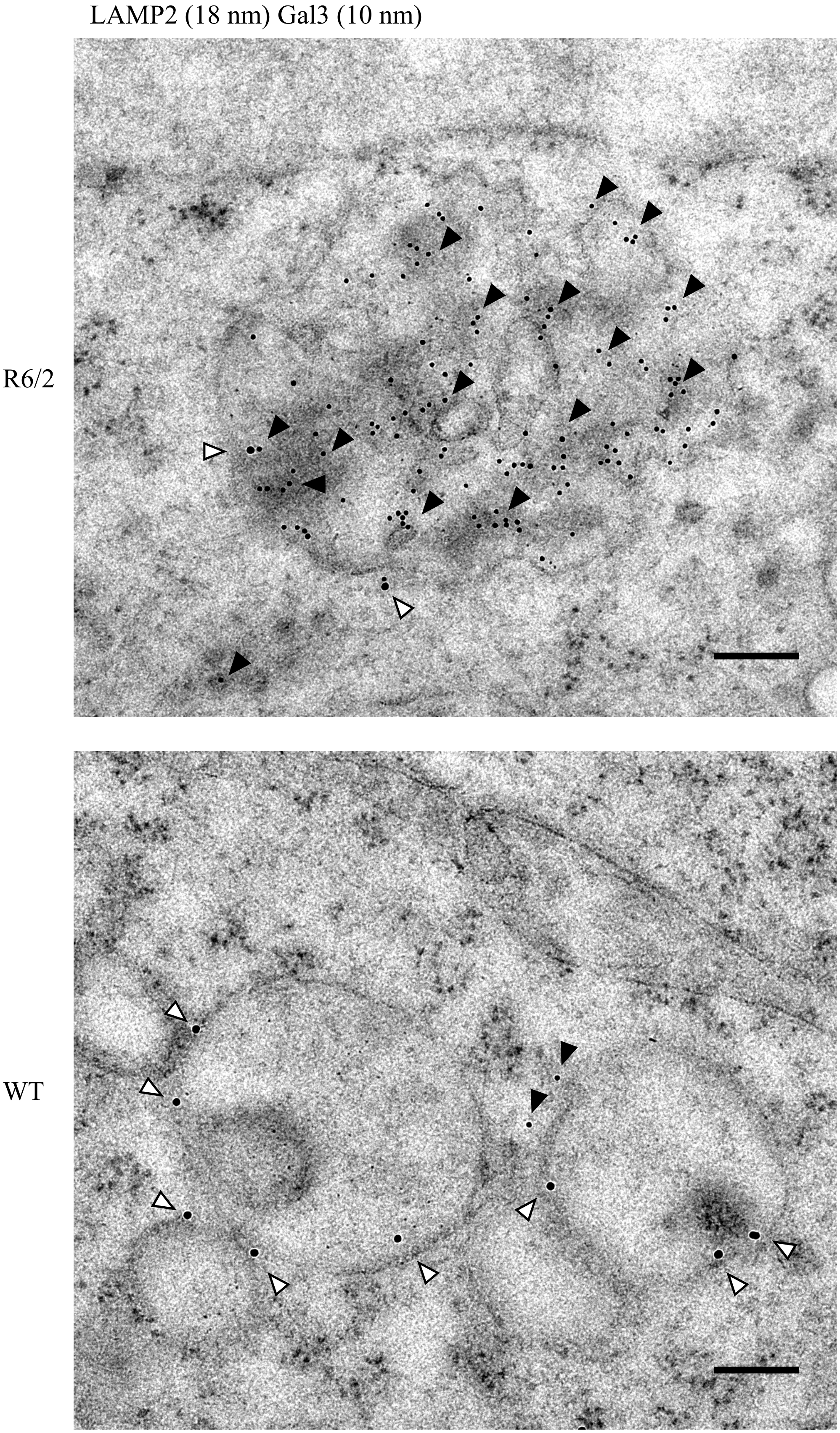

Supplementary Figure 9. Gal3 accumulates in lysosome-like structures of R6/2 microglia. Immunogold labeling of WT and R6/2 primary microglia with antibodies against lysosomes (LAMP2) and Gal3. Lysosome-like structures were identified by staining for a lysosomal marker, LAMP2 (18 nm, white arrows) and Gal3 localization was demonstrated by using 10 nm gold particles (black arrows). For the sake of simplicity, only a portion of Gal3 gold particles are marked with black arrows in the lysosome of R6/2 microglia. Scale bar: 0.2 μm.

Supplementary Fig. 10 (Related to Fig. 6)

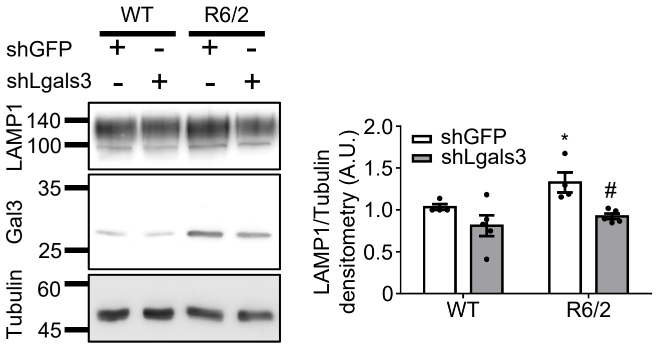

Supplementary Figure 10. Suppression of Gal3 improves the clearance of damaged lysosomes. Primary microglia were infected with the indicated lentiviruses expressing shRNA against Gal3 (i.e., sh*Lgals3*) to knock down Gal3 or the control lentivirus (sh*GFP*). One week after infection, the levels of Gal3 and LAMP1 were analyzed by Western blot. The results were analyzed by two-way ANOVA, followed by the Newman-Keuls post hoc test. Data are presented as the means ± SEM. *, Specific comparison between WT and R6/2 cells infected with the same lentivirus; #, Specific comparison between the sh*GFP*- and sh*Lgals3*-infected groups of the same genotype; *$P < 0.05$. Same *P*-value denotation for #.

Supplementary Fig. 11 (Related to Fig. 7)

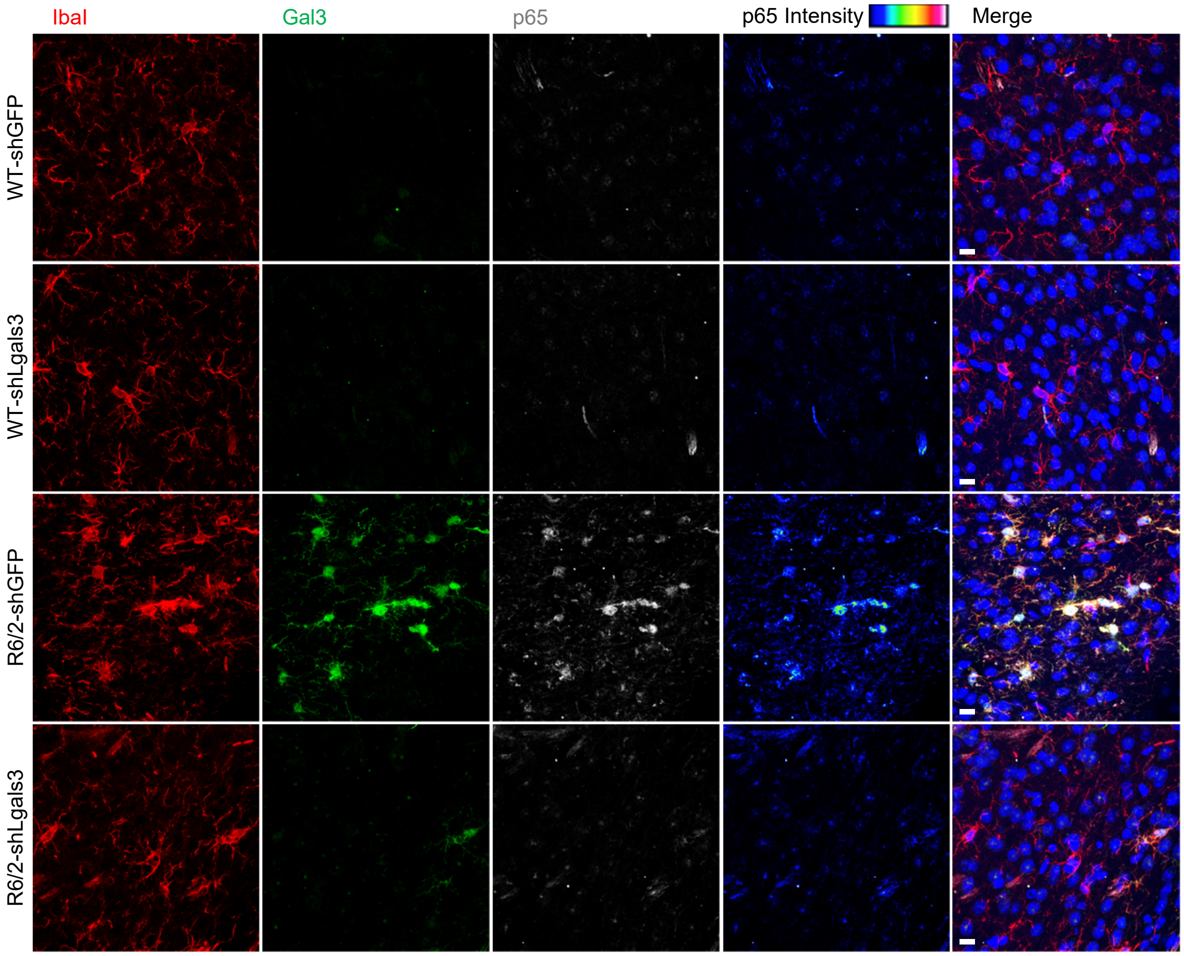

Supplementary Figure 11. Knockdown of Gal3 in the brains of R6/2 mice reduces the activation of microglia. Mice of 6 weeks were intrastriatally injected with lentiviruses carrying the indicated shRNA and monitored for additional 7 weeks. Brain tissues were carefully harvested and subjected to immunofluorescence assay using the indicated antibodies. Nuclei were stained with Hoechst (blue). The expression of Gal3 (green), IbaI (red), and p65 (gray) in the striatum of the indicated mouse were assessed. The color bars labeled as p65 intensity represent the level of p65 intensity, from low to high fluorescence signals (blue → red). Twelve image frames of each animal were analyzed (4 animals in each group). Representative images are shown. Scale bar: 10 µm.

Supplementary Fig. 12 (Related to Fig. 7)

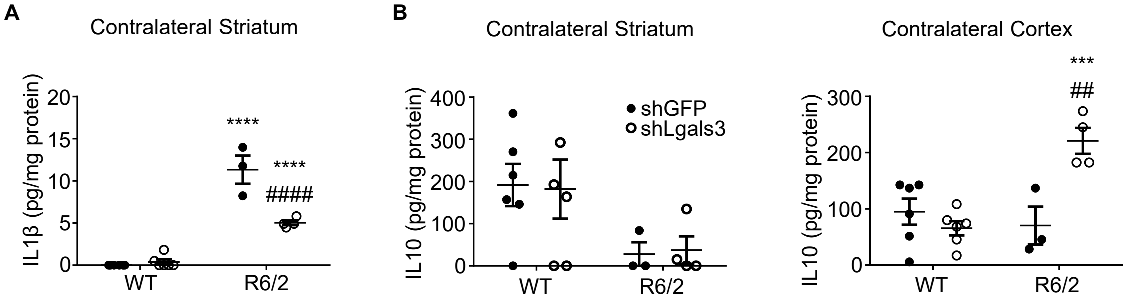

Supplementary Figure 12. Knockdown of Gal3 in the brains of R6/2 mice reduces inflammation. Mice of 6 weeks were intrastriatally injected with lentiviruses carrying the indicated shRNA and monitored for additional 7 weeks. Homogenates prepared from the non-injected/contralateral striatum were analyzed for the levels of **(A)** IL1β and **(B)** IL10 by ELISA. Each dot represents the mean value of each mouse (3 – 6 animals in each group). Data are presented as the means ± SEM. Results were analyzed by two-way ANOVA followed by Tukey's post hoc test. *, Specific comparison between WT and R6/2 mice of infected with the same lentivirus; #, Specific comparison between mice of the same genotype. $^{**}P < 0.01$, $^{***}P < 0.001$, $^{****}P < 0.0001$. Same *P*-value denotation for #.

Supplementary Fig. 13 (Related to Fig. 7 & 8)

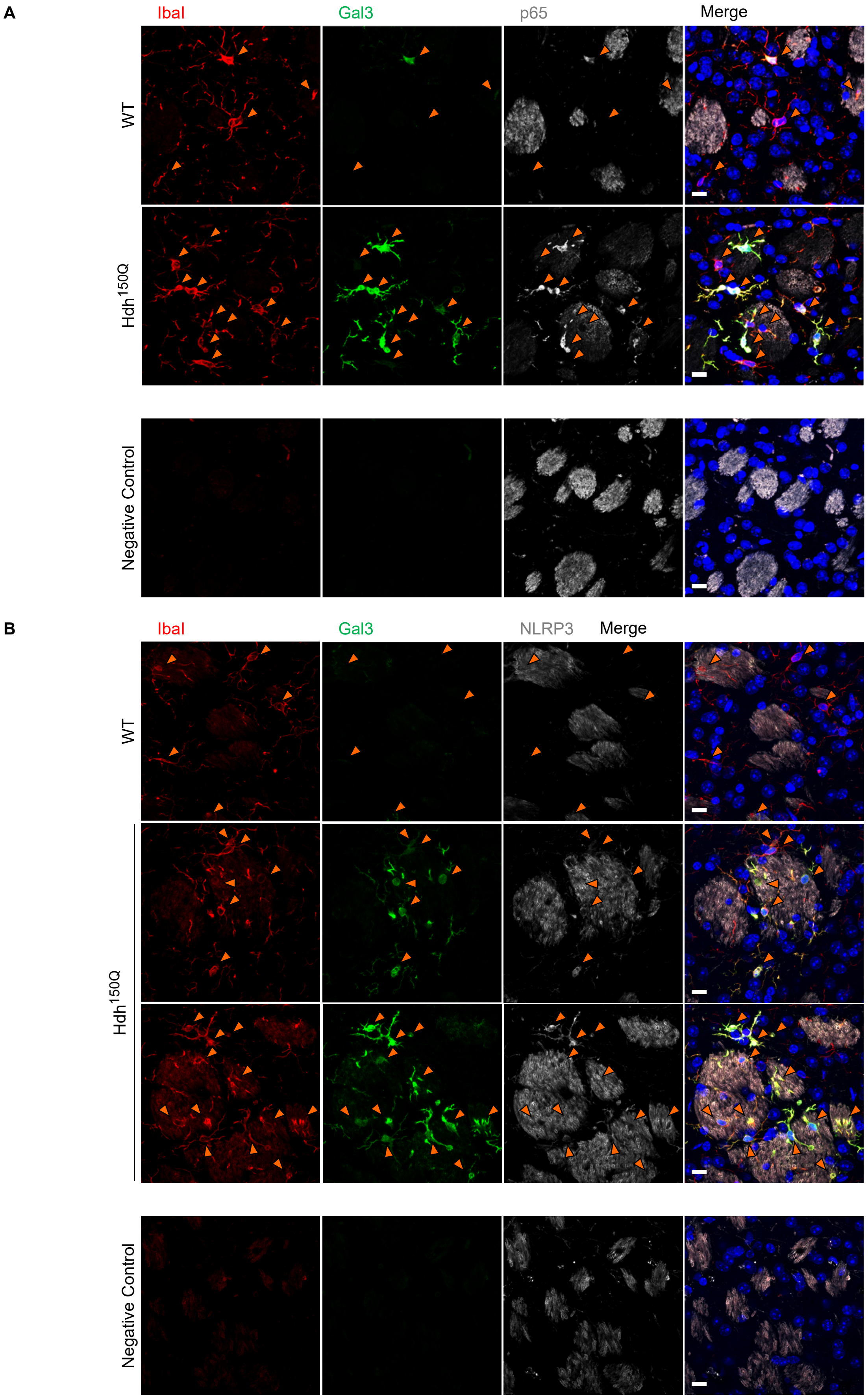

Supplementary Figure 13. Activation of NFκB and NLRP3 in microglia of Hdh^{150Q} mice. Brain sections from WT and Hdh^{150Q} mice (21 months old, n = 3 in each group) were stained with a Gal3 antibody (green), a microglial marker (IbaI, red) and **(A)** p65 (gray) or **(B)** NLRP3 (gray). Nuclei were stained with Hoechst (blue). Arrows mark the IbaI-positive cells (i.e., microglia). Negative controls were stained with secondary antibodies without respective primary antibodies. Scale bar: 10 μm.

Supplementary Fig. 14

Fig. 2C

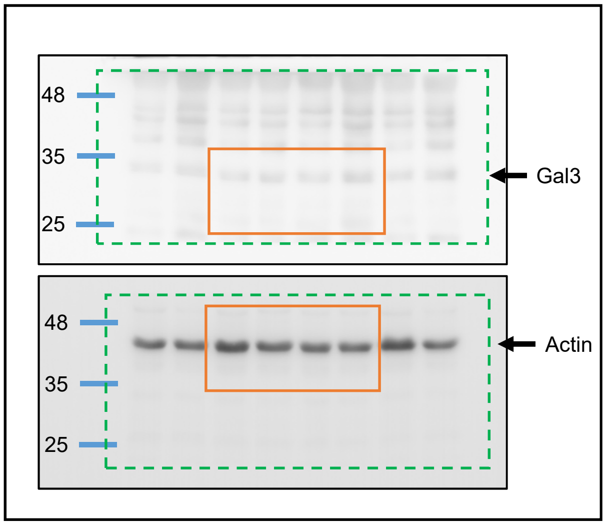

Supplementary Fig. 5A

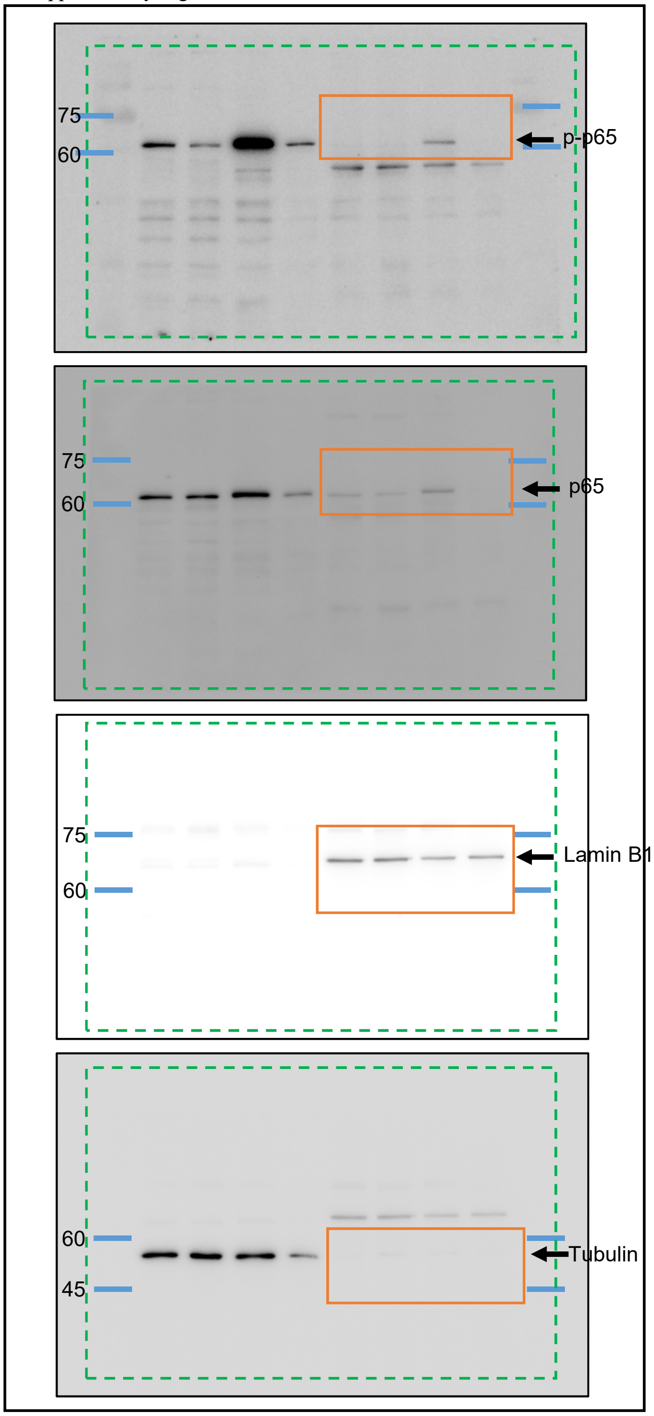

Supplementary Fig. 5B

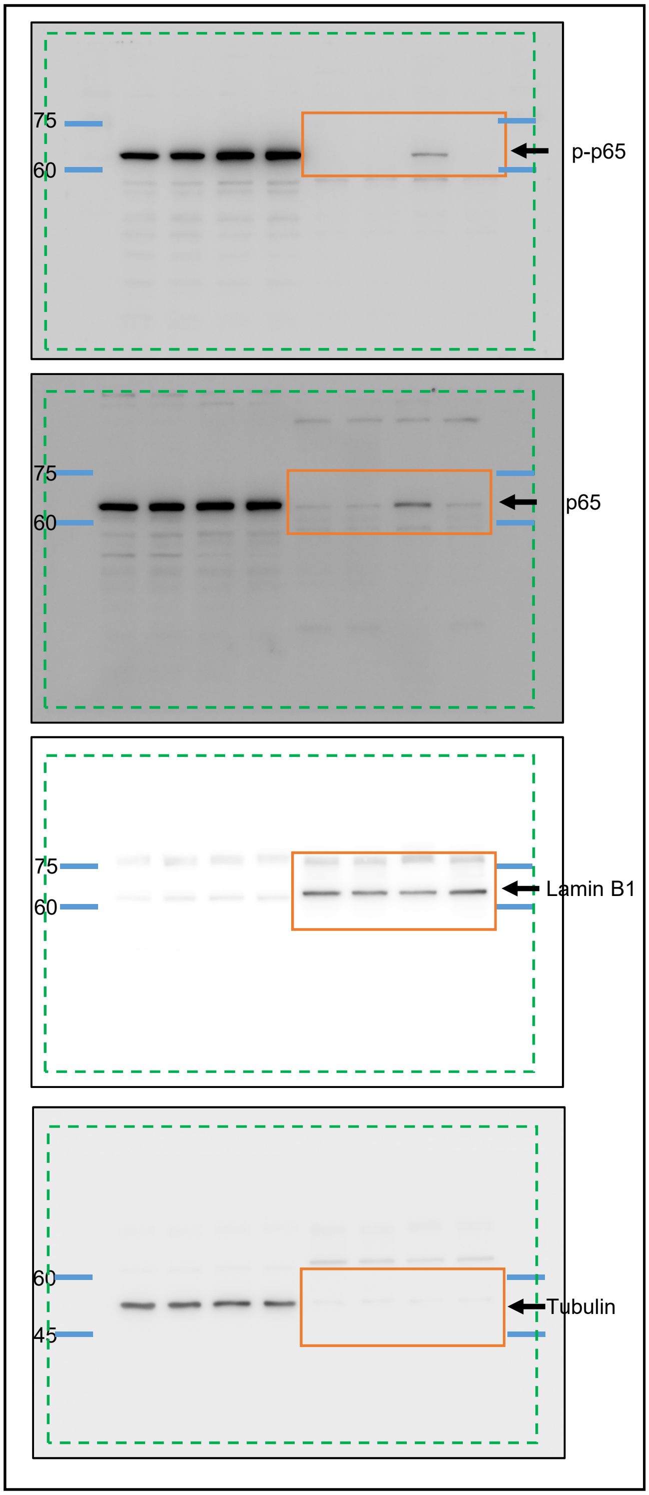

(see caption on the next page)

Supplementary Fig. 14

Supplementary Fig. 10

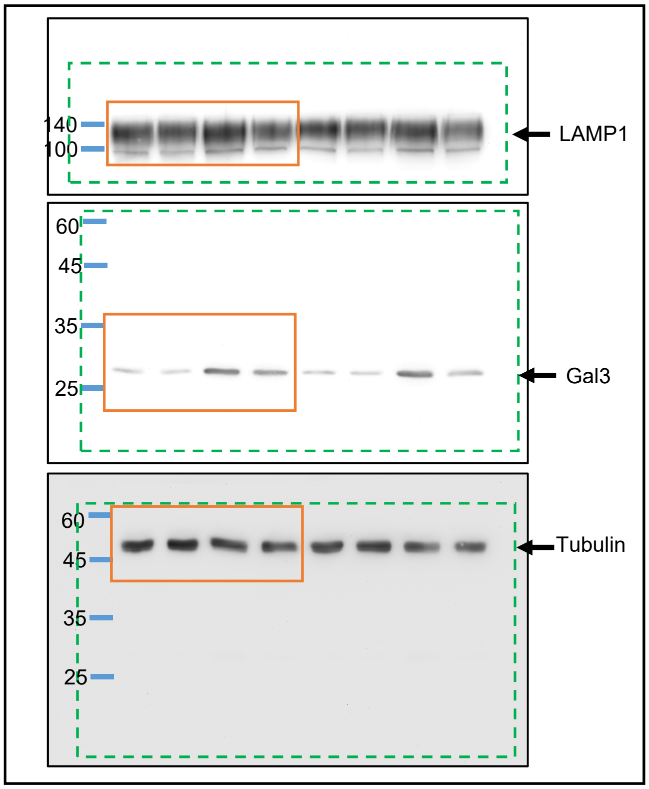

Supplementary Figure 14. *(include image on the previous page)*
Uncropped data for immunoblots. The cropped regions are indicated by orange lines, molecular weight markers are indicated by blue lines and perimeters of PVDF membranes are indicated by dashes green lines.

Supplementary Fig. 15 (Related to Fig. 2)

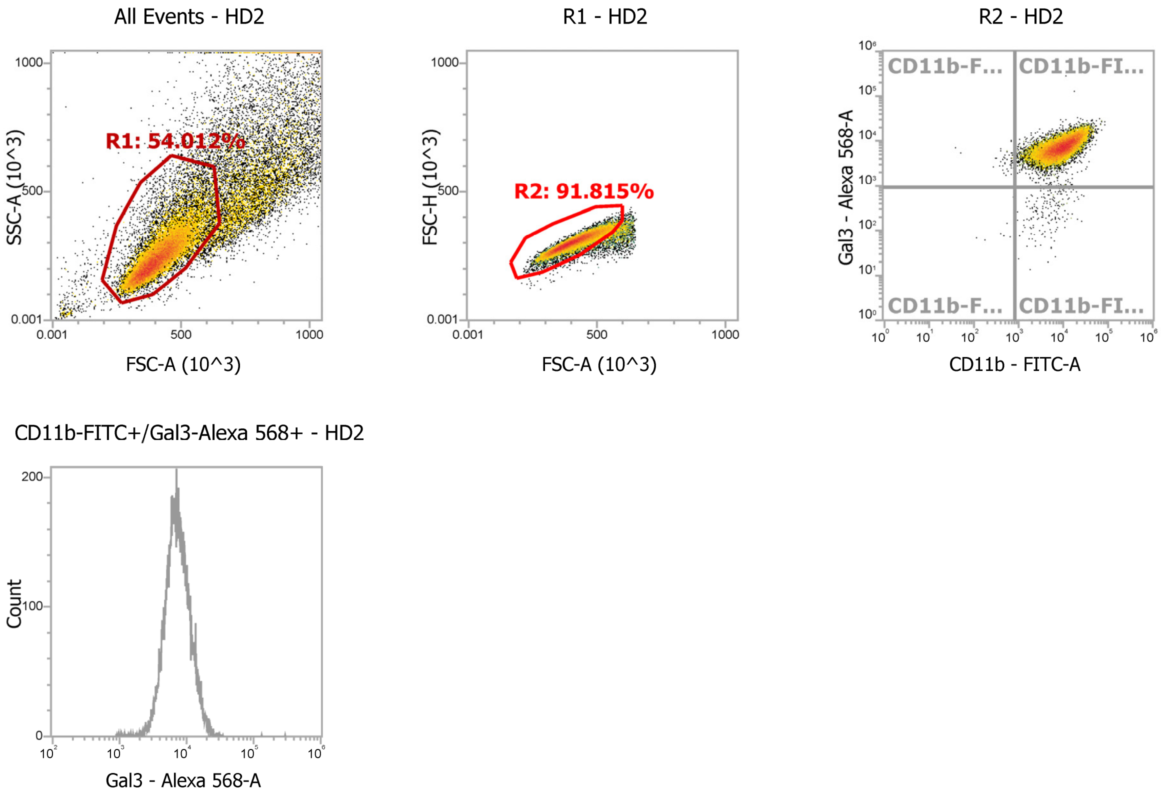

Supplementary Figure 15. Gating strategy used for flow cytometry. Gating strategy for detection of Gal3-Alexa 568 and CD11b-FITC.

Supplementary Tables

Supplementary Table 1: (Related to Fig. 1)

Clinical demographic data of Huntington's disease (HD) patients.

	Non-HD control	HD
Mean age (years)	51.2 ± 12.5	50.6 ± 12.6
Gender	7 men and 9 women	14 men and 16 women
Mean onset age (years)	-	45.1 ± 12.7
Disease duration (years)	-	8.0 ± 8.7
CAG repeats	-	44 ± 3

Data were presented as the means ± SD.

Supplementary Table 2: (Related to Fig. 1)

Brain tissue specimens from NIH NeuroBioBank (USA) for RNA preparation and qPCR analysis.

Case	Neuropath Diagnosis	Clinical Diagnosis	Tissue	Age (years)	Gender	PMI (hour)
2691	HD	HD, Depression	Caudate putamen, cerebellum	65	Female	13.0
2706	HD	HD	Caudate putamen, cerebellum	43	Male	17.0
3091	HD	HD	Caudate putamen, cerebellum	44	Female	9.0
3358	HD	HD	Caudate putamen, cerebellum	45	Female	8.7
3881	HD	HD	Caudate putamen, cerebellum	65	Male	9.1
4332	Normal	Colon cancer with metastasis	Caudate putamen, cerebellum	63	Male	12.0
4431	Normal	Myocardial infarction	Caudate putamen, cerebellum	68	Female	23.7
4631	Normal	Chronic obstructive pulmonary disease, Emphysema, Congestive heart failure, Hypertension	Caudate putamen, cerebellum	59	Male	20.2
4716	Normal, incidental age related changes	Alzheimer disease (Early Onset), Seizure, Dementia, Possible stroke	Caudate putamen, cerebellum	55	Female	19.7
3805	Normal	Renal Failure, Acute Diabetes mellitus	Caudate putamen, cerebellum	70	Male	12.0

Supplementary Table 3: (Related to Supplementary Fig. 1)

Brain tissue specimens from NIH NeuroBioBank (USA) for immunochemical staining analysis.

Case	Neuropath Diagnosis	Clinical Diagnosis	Tissue	Age (years)	Gender	PMI (hour)
5413	HD	Not determined	Caudate putamen	43	Male	10
5217	HD	Not determined	Caudate putamen	55	Male	10
S04231	HD	Atherosclerosis and arteriosclerosis	Caudate putamen	68	Male	13.16
S14445	HD	Arteriosclerosis	Caudate putamen	68	Female	17
M4042M	Non-HD	Not determined	Caudate putamen	58	Male	9
S04888	Non-HD	Arteriosclerosis, mild	Caudate putamen	58	Male	19.05
S12237	Non-HD	Normal control	Caudate putamen	68	Male	16.05

Supplementary Table 4:

List of primers used for genotyping and amplification of transcripts in qPCR analyses.

**Gene name**	**Forward primer (5'-3')**	**Reverse primer (5'-3')**
R6/2-genotyping	CCGCTCAGGTTCTGCTTTTA	GGCTGAGGAAGCTGAGGAG
Hdh^{150Q}-genotyping	CCCATTCATTGCCTTGCTG	GCGGCTGAGGGGGTTGA
LGALS3	CAGAATTGCTTTAGATTTCCAA	TTATCCAGCTTTGTATTGCAA
Lgals3	TTGAAGCTGACCACTTCAAGGTT	AGGTTCTTCATCCGATGGTTGT
18s	GTAACCCGTTGAACCCCATT	CCATCCAATCGGTAGTAGCG
Gapdh	TGACATCAAGAAGGTGGTGAAG	AGAGTGGGAGTTGCTGTTGAAG

Supplementary Methods

Immunofluorescence staining of human brain sections. Human brain tissue specimens were obtained from the NIH NeuroBioBank (USA). Supplementary Table 3 summarizes the specimen demographic data. The brain sections (5 μm) were treated with an antigen retrieval reagent (Sigma; pH 6.0) at 97.5°C for 20 min, washed with 0.1 M Na-PBS, and permeabilized with 0.1 M Na-PBS containing 0.2% Triton X-100 for 10 min. The sections were then blocked with 4% BSA for 2 hours at room temperature. The specimens were incubated with the indicated primary antibody at 4°C in a humidified chamber for 2 days, followed by 2 hours of incubation with the corresponding secondary antibody at room temperature (RT). The primary antibodies included anti-Iba1 (019-19741, Wako Laboratory Chemicals) and anti-Gal3 (556904, BD Pharmingen). After extensive washes, the brain sections were treated with 0.1% (w/v) Sudan Black B (Sigma) in 70% ethanol for 15 min at RT before nuclear staining to block autofluorescence signal. Nuclei were stained with Hoechst 33258. The slides were then analyzed by confocal microscopy (LSM780 microscope and Zen 2012 software; Carl Zeiss, Germany). For imaging, 7 z-stacks were taken for each image, with a thickness of 5 μm. The settings (z-stacks and thickness) were identical when taking images for the same experiment.

Transmission electron microscopy (TEM) and immunoelectron microscopy. TEM was carried out using protocols as detailed elsewhere with slight modifications [1]. Briefly, cells were fixed overnight with 4% paraformaldehyde plus 2.5% glutaraldehyde in PBS. After washing with PBS, the specimens were fixed with 1% osmium tetroxide (OsO_4) in PBS for 2 hours. The specimens were washed with PBS, followed by dehydration with ethanol and acetone at room temperature. For immunoelectron microscopy, sample were prepared according to protocols detailed elsewhere with slight modification [2]. Specimens were subjected to high-pressure freezing in 1-hexadecene/20%

BSA by a Leica EM HPM100. After that, specimens were subjected to freeze substitution for fixation and staining with 0.1% uranyl acetate and acetone in a Leica EM AFS2 according to the standard protocol. The specimens were then subjected to resin infiltration with a combination of acetone and Spurr's resin at ratios of 3:1 for 4 hours, 1:1 for 4 hours, and 1:3 overnight, and followed by incubation with pure Spurr's resin overnight. The specimens were then transferred to fresh pure resin for 4 hours before polymerization in an oven at 60°C for 48 hours. The specimens were sectioned by using an ultramicrotome (Leica EM UC7, Gmbh). For immunoelectron microscopy, the specimens were blocked with 5% BSA and 5% NGS for 30 min at room temperature. Specimens were stained with an anti-Gal3 [3] and an anti-LAMP2 antibodies (ab25339, Abcam) overnight at 4°C. After that, the specimens were washed with 5 droplets of 0.1% BSA/NGS in PBS and labeled with respective gold particle-conjugated secondary antibodies for 1 hr at room temperature. The specimens were then washed with 5 droplets of 0.1% BSA/NGS in PBS, PBS and ddH_2O. The specimens were observed and photographed under an FEI Tecnai G2 F20 S-Twin electron microscope (FEI, USA).

Primary microglia from adult mice and treatments. Twelve-week-old mice were anesthetized and perfused with ice-cold saline (0.9% (w/v) NaCl). Brains were carefully dissected to obtain the cortex and striatum. The collected tissues were pooled from two brains and were enzymatically digested to single cells using a Neural Tissues Dissociation Kit (Miltenyi Biotec, Gmbh) according to the manufacturer's instructions. Microglia were isolated from the cell mixture as described elsewhere [4]. In brief, the cell pellets were resuspended in 4 ml of 37% Percoll solution and transferred to a 15-ml tube, which was subsequently underlaid with 4 ml of 70% Percoll. We then slowly added 4 ml of 30% Percoll on top of the 37% Percoll, followed by 2 ml of HBSS. The samples were subjected to centrifugation at 300 x g for 40 min with no brake. Microglia, were

collected at the 70%-37% interphase layer and were collected and cultured on poly-L-lysine-coated coverslips in DMEM/F12 (Invitrogen, Grand Island, USA) supplemented with 10% FBS, 2 mM L-glutamine and 1% penicillin/streptomycin at 37°C in a humidified 5% CO_2-containing atmosphere. The purity of isolated microglia was determined by immunocytochemistry using anti-Iba1, anti-GFAP and anti-NeuN antibodies. For experimentation, after 24 hours of plating, we replaced the culture medium with fresh medium containing TD139 (20 μM) or DMSO for another 24 hours. Cellular debris would have been removed during the changing of medium. After that, culture medium was collected, and cells were fixed for further analysis. All comparative experiments between HD and WT groups were carried out using microglia isolated and cultured simultaneously for 2 days under the same conditions.

Supplementary References

1 Graham, L. & Orenstein, J. M. Processing tissue and cells for transmission electron microscopy in diagnostic pathology and research. *Nat Protoc* **2**, 2439-2450, doi:10.1038/nprot.2007.304 (2007).

2 Studer, D. *et al.* Capture of activity-induced ultrastructural changes at synapses by high-pressure freezing of brain tissue. *Nat Protoc* **9**, 1480-1495, doi:10.1038/nprot.2014.099 (2014).

3 Liu, F. T. *et al.* Expression and function of galectin-3, a beta-galactoside-binding lectin, in human monocytes and macrophages. *Am J Pathol* **147**, 1016-1028 (1995).

4 Lee, J. K. & Tansey, M. G. Microglia isolation from adult mouse brain. *Methods Mol Biol* **1041**, 17-23, doi:10.1007/978-1-62703-520-0_3 (2013).
