# Supplementary material for: Galectin-3 is required for the microglia-mediated brain inflammation in a model of Huntington’s disease
Source: Nat Commun. 2019 Aug 2;10:3473. doi: 10.1038/s41467-019-11441-0 (PMC6677843; doi:10.1038/s41467-019-11441-0)
Supplement: Supplementary file 3 — Supplementary Software 1 [file 41467_2019_11441_MOESM3_ESM.pdf]

**Supplementary Software 1 METAMORPH**

**Imaging processing and quantification:**

1. Open file.

2. For image containing far red channel >Edit> Duplicate plane (all plane for analysis)
3. For RGB image >Display>Colour Separate

4. Process> Arithmetic
   - Source image 1: All image/plane from previous step
   - Source 2: Constant value
   - Result section – Image: Add
   - Bit depth: 16 bit
   - Operation: Add
   - Constant value: 0 gray levels
   - New image file will be generated (Add-1, Add-2 …)

5. Apps> Multi wavelength cell scoring
   - Number of wavelengths: 4 (depending on the image containing how many channels, channel-1 (Add-1) always for All nuclei)
   - Display result image: Segmentation (Check)
   - Select source image according the channel arrangement (Add-1, Add-2 …)
   - Stain area: Select nucleus, cytoplasm or both
   - Scoring parameter:
     - Min width: *refer to each figure setting* µm
     - Max width: *refer to each figure setting* µm
     - Intensity above local background: *refer to each figure setting* graylevels

6. Configure Data Log (Parameter configuration)
   - Image name
   - Cell: Assigned Label#
   - Cell: Total Area
   - Cell: Positive W2
   - Cell: W2 Stained Area
   - Cell: W2 Stained Integr Intensity
   - Cell: W2 Stained Average Intensity
   - Cell: W2 Nucleus Integr Intensity (*Optional*)
   - Cell: W2 Nucleus Average Intensity (*Optional*)
   - Cell: W2 Cytoplasm Integr Intensity (*Optional*)
   - Cell: W2 Cytoplasm Average Intensity (*Optional*)

   Same parameter configuration for other wavelengths (i.e., W3, W4)

Log column titles (check)

7. For tissue sample images, IbaI signals are used to identify microglia.

- Example, IbaI is represented by wavelength 2 (W2), under the Cell: Positive W2 column, “0” value will be filtered, and the remaining column with “1” value represent microglia (“0” = W2 (IbaI) – negative cells; “1” = W2 (IbaI) – positive cells).

**Puncta quantification:**

1. Open image file.

2. Save files as Meta Single/Multiple Plane TIFF.

3. Edit> Duplicate > all plane

4. Process> Morphology Filters

- Source image: select puncta plane
- Result image: Top Hat
- Operation: Extract features > Top hat
- Filter shape: Circle
- Diameter 10 pixels
- Reconstruction (check)

5. Process> Arithmetic

- Source image 1: Select nucleus frame
- Source image 2: Constant value
- Result > Image > Add
- Bit depth: 16 bit
- Operation: Add
- Constant values: 0

6. Repeat step 5 on Top Hat generated puncta plane in Step 4.

7. Repeat step 5 on positive marker plane.

8. Apps> Cell scoring

- W1 Source image: Nuclear image generated from Step 5 (i.e., Add)
  - Min width: 6
  - Max width: 12
  - Intensity above local background: 8 gray levels
- W2 Source image: Positive marker image generated from Step 5 (i.e., Add-3)
  - Stained area: Cytoplasm and nucleus
  - Min width: 5
  - Max width: 40
  - Intensity above local background: 20 gray levels
- Display result image: Segmentation (check)

9. Process> Binary operations

- Source image: Segmentation generated in Step 8
- Result image: Binary
- Operation >Binarize

- Parameters: 1 – 3

10. Process> Arithmetic

- Source image 1: puncta plane generated in Step 5 (i.e., Add-2)
- Source image 2: Binary generated at Step 9
- Result image: AND
- Bit depth: 16 bit
- Operation: Logical AND

11. Apps> Granularity

- Granule image: AND generated from Step 10
    - Display result image: Granules (check)
    - Min width: 0.8
    - Max width: 1.3
    - Intensity above local background: 100
- Nuclear image: nuclear plane generated from Step 5 (i.e., Add)
    - Min width: 6
    - Max width: 12
    - Intensity above local background: 5

12. Configure Data Log (Cells)

- Image Name
- Cell: Assigned Label #
- Cell: Granule Count
- Cell: Granule Total Area
- Cell: Granule Integrated Intensity
- Cell: Granule Average Intensity

**Colocalization quantification:**

1. Open file.

2. For image containing far red channel >Edit> Duplicate plane (all plane for analysis)
3. For RGB image >Display>Colour Separate

4. Process> Arithmetic
   - Source image 1: All image/plane from previous step
   - Source 2: Constant value
   - Result section – Image: Add
   - Bit depth: 16 bit
   - Operation: Add
   - Constant value: 0 gray levels

5. New image file will be generated (Add-1, Add-2 …)

6. Process> Morphology Filters
   - Source image: select planes to measure
   - Result image: Top Hat
   - Operation: Extract features > Top hat
   - Filter shape: Circle
   - Diameter: For LAMP1 > 5 pixels > Use reconstruction (check)<br>Gal3 > 5 pixels > Use reconstruction (check)
   - Reconstruction (check)

7. Measure> Threshold Image
   - Inclusive LAMP1: 15-255
   - Inclusive Gal3: 20-255

8. Apps>Colocalization

**Quantification Parameters:**

(*Unless stated otherwise, W1, W2, W3 and W4 indicates blue, green, red and far red fluorescence signal, respectively.*)

Fig. 2D

Scoring parameter:

| | **W1** | **W2** | **W3** | **W4** |
|---|---|---|---|---|
| Min width | 5 | 3 | 3 | - |
| Max width | 15 | 100 | 100 | - |
| Intensity above local background | 5 | 11 | 25 | - |
| Minimum stained area | - | 10 | 10 | - |

Fig. 2F

| | **W1** | **W2** | **W3** | **W4** |
|---|---|---|---|---|
| Min width | 5 | 5 | - | 5 |
| Max width | 24 | 24 | - | 24 |
| Intensity above local background | 40 | 4 | - | 4 |
| Minimum stained area | - | 40 | - | 40 |

Fig. 2J

| | **W1** | **W2** | **W3** | **W4** |
|---|---|---|---|---|
| Min width | 4 | 4 | 2 | - |
| Max width | 30 | 30 | 30 | - |
| Intensity above local background | 15 | 20 | 90 | - |
| Minimum stained area | - | 5 | 5 | - |

Fig. 3A

| | **W1** | **W2** | **W3** | **W4** |
|---|---|---|---|---|
| Min width | 5 | 5 | 5 | 5 |
| Max width | 20 | 20 | 20 | 20 |
| Intensity above local background | 50 | 20 | 20 | 20 |
| Minimum stained area | - | 100 | 100 | 100 |

Fig. 4A

| | **W1** | **W2** | **W3** | **W4** |
|---|---|---|---|---|
| Min width | 5 | 5 | 5 | 5 |
| Max width | 20 | 20 | 20 | 20 |
| Intensity above local background | 50 | 20 | 20 | 20 |
| Minimum stained area | - | 100 | 100 | 100 |

Fig. 6B

Cell scoring

| | **W1** | **W2** | **W3** | **W4** |
|---|---|---|---|---|
| Min width | 6 | 5 | - | - |
| Max width | 12 | 40 | - | - |
| Intensity above local background | 8 | 20 | - | - |
| Stained area | - | Cytoplasm and nucleus | - | - |

Granularity

| | **Granule image** | **Nuclear image** | **-** | **-** |
|---|---|---|---|---|
| Min width | 0.8 | 6 | - | - |
| Max width | 1.3 | 12 | - | - |
| Intensity above local background | 100 | 5 | - | - |

Fig. 6C

| | **W1** | **W2** | **W3** | **W4** |
|---|---|---|---|---|
| Min width | 5 | 5 | 3 | - |
| Max width | 15 | 50 | 50 | - |
| Intensity above local background | 40 | 5 | 5 | - |
| Minimum stained area | - | 10 | 10 | - |

Fig. 6D

| | **W1** | **W2** | **W3** | **W4** |
|---|---|---|---|---|

| | | | | |
|---|---|---|---|---|
| Min width | 6 | 5 | 3 | 3 |
| Max width | 15 | 50 | 50 | 50 |
| Intensity above local background | 40 | 5 | 8 | 8 |
| Minimum stained area | - | 10 | 10 | 10 |

Fig. 6E

| | **W1** | **W2 (red)** | **W3** | **W4** |
|---|---|---|---|---|
| Min width | 5 | 2 | - | - |
| Max width | 20 | 20 | - | - |
| Intensity above local background | 50 | 15 | - | - |
| Minimum stained area | - | 5 | - | - |

Fig. 7A

| | **W1** | **W2** | **W3** | **W4** |
|---|---|---|---|---|
| Min width | 5.5 | 1 | 1 | 1 |
| Max width | 12 | 20 | 20 | 20 |
| Intensity above local background | 16 | 40 | 40 | 90 |
| Minimum stained area | - | 30 | 30 | 30 |

Fig. 7B Supplementary Fig. 7

| | **W1** | **W2** | **W3** | **W4** |
|---|---|---|---|---|
| Min width | 5.5 | 1 | 1 | 1 |
| Max width | 12 | 20 | 20 | 20 |
| Intensity above local background | 16 | 40 | 40 | 90 |
| Minimum stained area | - | 30 | 30 | 30 |

Fig. 8A

| | **W1** | **W2** | **W3** | **W4** |
|---|---|---|---|---|
| Min width | 5.5 | 1 | 1 | 1 |
| Max width | 15 | 20 | 20 | 20 |
| Intensity above local background | 10 | 40 | 40 | 90 |

| | | | | |
|---|---|---|---|---|
| Minimum stained area | - | 30 | 30 | 30 |

Fig. 9D

| | W1 | W2 | W3 | W4 |
|---|---|---|---|---|
| Min width | 5 | 1 | 5 | - |
| Max width | 10 | 7 | 10 | - |
| Intensity above local background | 5 | 30 | 5 | - |
| Minimum stained area | - | 5 | 30 | - |

Fig. 9D - 2

Cell scoring

| | W1 | W2 | W3 | W4 |
|---|---|---|---|---|
| Min width | 4 | 5 | - | - |
| Max width | 6 | 6 | - | - |
| Intensity above local background | 5 | 18 | - | - |
| Stained area | - | Nucleus | - | - |

Granularity

| | Granule image | Nuclear image | - | - |
|---|---|---|---|---|
| Min width | 1.5 | 5 | - | - |
| Max width | 7 | 6 | - | - |
| Intensity above local background | 70/20 | 18 | - | - |

Fig. 9G

| | W1 | W2 | W3 | W4 |
|---|---|---|---|---|
| Min width | 5 | 5 | 5 | - |
| Max width | 8 | 50 | 15 | - |
| Intensity above local background | 5 | 10 | 18 | - |
| Minimum stained area | - | 50 | 50 | - |

Fig. 9G - 2

| | W1 | W2 | W3 | W4 |
|---|---|---|---|---|
| Min width | 5 | 3 | 3 | |
| Max width | 8 | 40 | 40 | |
| Intensity above local background | 5 | 5 | 15 | |
| Minimum stained area | - | 50 | 50 | |

Supplementary Fig. 3A

| | W1 | W2 | W3 | W4 |
|---|---|---|---|---|
| Min width | 4 | 10 | 7 | - |
| Max width | 8 | 30 | 40 | - |
| Intensity above local background | 20 | 6 | 5 | - |
| Minimum stained area | - | 10 | 10 | - |

Supplementary Fig. 4

| | W1 | W2 | W3 | W4 |
|---|---|---|---|---|
| Min width | 6 | 3 | 3 | - |
| Max width | 30 | 20 | 20 | - |
| Intensity above local background | 20 | 15 | 20 | - |
| Minimum stained area | - | 3 | 1 | - |

Supplementary Fig. 6A

| | W1 | W2 | W3 | W4 |
|---|---|---|---|---|
| Min width | 5 | 10 | 10 | - |
| Max width | 10 | 40 | 40 | - |
| Intensity above local background | 5 | 7 | 7 | - |
| Minimum stained area | - | 10 | 10 | - |
