# Supplementary material for: Galectin-3 is required for the microglia-mediated brain inflammation in a model of Huntington’s disease
Source: Nat Commun. 2019 Aug 2;10:3473. doi: 10.1038/s41467-019-11441-0 (PMC6677843; doi:10.1038/s41467-019-11441-0)
Supplement: Supplementary file 4 — Reporting Summary [file 41467_2019_11441_MOESM4_ESM.pdf]

nature research

Corresponding author(s): Yijuang Chern

Last updated by author(s): Jul 5, 2019

Reporting Summary

Nature Research wishes to improve the reproducibility of the work that we publish. This form provides structure for consistency and transparency in reporting. For further information on Nature Research policies, see Authors & Referees and the Editorial Policy Checklist.

Statistics

For all statistical analyses, confirm that the following items are present in the figure legend, table legend, main text, or Methods section.

n/a	Confirmed	
☐	☒	The exact sample size (*n*) for each experimental group/condition, given as a discrete number and unit of measurement
☐	☒	A statement on whether measurements were taken from distinct samples or whether the same sample was measured repeatedly
☐	☒	The statistical test(s) used AND whether they are one- or two-sided<br>*Only common tests should be described solely by name; describe more complex techniques in the Methods section.*
☐	☒	A description of all covariates tested
☐	☒	A description of any assumptions or corrections, such as tests of normality and adjustment for multiple comparisons
☐	☒	A full description of the statistical parameters including central tendency (e.g. means) or other basic estimates (e.g. regression coefficient) AND variation (e.g. standard deviation) or associated estimates of uncertainty (e.g. confidence intervals)
☒	☐	For null hypothesis testing, the test statistic (e.g. *F*, *t*, *r*) with confidence intervals, effect sizes, degrees of freedom and *P* value noted<br>*Give P values as exact values whenever suitable.*
☒	☐	For Bayesian analysis, information on the choice of priors and Markov chain Monte Carlo settings
☒	☐	For hierarchical and complex designs, identification of the appropriate level for tests and full reporting of outcomes
☒	☐	Estimates of effect sizes (e.g. Cohen's *d*, Pearson's *r*), indicating how they were calculated

Our web collection on statistics for biologists contains articles on many of the points above.

Software and code

Policy information about availability of computer code

Data collection	LSM780 - Zen 2011 (SP6 64 bit Black edition Version 13.0)<br>LSM700 - Zen 2012 (SP1 64 bit Black edition Version 8.1)<br>ABI 7900HT - SDS 2.3<br>LightCycler 480 Software (SP3 Version 1.5.1.62)<br>Attune NxT Software Version 2.7.0
Data analysis	Zen 2.3 (SP1 64 bit Black edition)<br>Zen 2.3 (SP1 64 bit Blue edition)<br>MetaMorph Offline (Version 7.7.5.0)<br>MetaMorph Offline (Version 7.6.5.0)<br>ABI 7900HT - SDS 2.2<br>LightCycler 480 Software (SP3 Version 1.5.1.62)<br>Attune NxT Software Version 2.7.0<br>ImageJ Version 1.47<br>GraphPad - PRISM 6

For manuscripts utilizing custom algorithms or software that are central to the research but not yet described in published literature, software must be made available to editors/reviewers. We strongly encourage code deposition in a community repository (e.g. GitHub). See the Nature Research guidelines for submitting code & software for further information.

Data

Policy information about availability of data

All manuscripts must include a data availability statement. This statement should provide the following information, where applicable:

- Accession codes, unique identifiers, or web links for publicly available datasets
- A list of figures that have associated raw data
- A description of any restrictions on data availability

All relevant data that support this study are available to any interested researches upon request to the corresponding author.

Field-specific reporting

Please select the one below that is the best fit for your research. If you are not sure, read the appropriate sections before making your selection.

☒ Life sciences ☐ Behavioural & social sciences ☐ Ecological, evolutionary & environmental sciences

For a reference copy of the document with all sections, see nature.com/documents/nr-reporting-summary-flat.pdf

Life sciences study design

All studies must disclose on these points even when the disclosure is negative.

Sample size	No statistical procedures were applied to pre-determine the sample sizes, but our numbers were similar to those generally practiced in the field.
Data exclusions	No data were excluded from the analyses.
Replication	All attempts at replication were successful.
Randomization	Age- and body weight- matched mice were allocated randomly into experimental groups. All other samples were allocated randomly into experimental groups.
Blinding	The investigators were blinded for human plasma Gal3 analysis. For primary microglia study, because the isolated cells were limited and required to be pooled together, hence, the genotype of the cells were known during the experimentation and the experimental conditions were not blinded to the investigators. Blinding for R6/2 mice were not relevant as the mice exhibited significant body weight loss and impaired motor function, which were absence in the WT mice.

Reporting for specific materials, systems and methods

We require information from authors about some types of materials, experimental systems and methods used in many studies. Here, indicate whether each material, system or method listed is relevant to your study. If you are not sure if a list item applies to your research, read the appropriate section before selecting a response.

Materials & experimental systems

n/a	Involved in the study	
☐	☒	Antibodies
☒	☐	Eukaryotic cell lines
☒	☐	Palaeontology
☐	☒	Animals and other organisms
☐	☒	Human research participants
☒	☐	Clinical data

Methods

n/a	Involved in the study	
☒	☐	ChIP-seq
☐	☒	Flow cytometry
☒	☐	MRI-based neuroimaging

Antibodies

Antibodies used

IbaI, 019-19741, Wako Laboratory Chemicals (1:500 for mouse and human brain sections staining, 1: 1,000 for cell samples staining)
IbaI, GTX632426, GeneTex (1:1,000 for cell samples staining)
Galectin-3 AF1197, R&D System (1: 300 for brain sections)
Mouse anti-Galectin-3 B2C10, 556904, BD Pharmingen (1: 300 for human brain sections staining)
Mouse anti-Galectin-3 B2C10 (provided by FT-Liu's lab) (0.5 µg/ml for cell samples staining)
Goat anti-Galectin-3 (provided by FT-Liu's lab) (1.0 µg/ml for cell samples staining)
Rabbit anti-Galectin-3 (provided by FT-Liu's lab) (1µg/ml for flow cytometry)
NFκB-p65, MAB3026, Millipore (1: 100 for tissue sections, 1: 200 for cell samples staining)
NFκB-p65, [112A1021] ab13594, Abcam (1: 1,000 for western blot)

Phospho-NFκB-p65 (SER536)(93H1), #3033 (Cell Signaling) (1: 1,000 for western blot)
LaminB1, GTX103292, GeneTex (1: 10,000 for western blot)
Tubulin, CP06, Calbiochem (1: 10,000 for western blot)
Actin, A2066, Sigma (1: 10,000 for western blot)
LAMP1, ab24170, Abcam (1: 500 for cell samples staining, 1: 1,000 for western blot)
LAMP2, ab25339, Abcam (1: 200 for cell samples staining, 1 : 50 for immunoelectron microscopy)
LC3, GTX127375, GeneTex (1: 2,000 for cell samples staining)
SDHB,GTX113833, GeneTex (1: 500 for cell samples staining)
EM48, MAB5374, Millipore (1: 500 for brain sections staining)
NeuN, ABN78, Millipore (1: 1,000 for brain sections staining)
NeuN, MAB377, Millipore (1: 1,000 for brain sections staining)
S100, Z0311, Dako (1: 1,000 for brain sections staining)
GFAP, G9269, Sigma (1: 1,000 for cell samples staining)
CD68, ab31630, Abcam (1: 100 for brain sections staining)
NLRP3, AG-20B-0014, AdipoGen (1: 200 for brain sections staining)
DARPP32, #2302, Cell Signaling (1: 50 for brain sections staining)
CD11b-FITC, 101205, BioLegend (1: 200 for flow cytometry)

Validation

IbaI, 019-19741, Wako Laboratory Chemicals
In manuscript: Fig. 2D, F, J, Fig. 3A, Fig. 4A, Fig. 7A, B, Fig. 8A, Supplementary Fig. 1, Supplementary Fig. 3A, Supplementary Fig. 4, Supplementary Fig. 6A, E, F, Supplementary Fig. 11, Supplementary Fig. 13.
-https://labchem-wako.fujifilm.com/us/category/01213.html
-Species Reactivity: mouse, rat, human
-Application: IHC, IF, ICC
-Nature 562(7728): 578-582.,2018

IbaI, GTX632426, GeneTex
In manuscript: Supplementary Fig. 3B, C, Supplementary Fig. 6E, F.
https://www.genetex.com/Product/Detail/Iba1-antibody-GT10312/GTX632426
-Species Reactivity: human, mouse, rat
-Application: WB, ICC/IF, IHC-P, IHC-Fr, FACS
-Nat Commun 10(1): 465., 2019

Galectin-3 AF1197, R&D System
In manuscript: Fig. 2D, J, Fig. 7A, B, 8A, Supplementary Fig. 2, Supplementary Fig. 11, 13.
-https://www.rndsystems.com/products/human-mouse-rat-galectin-3-antibody_af1197
-Species Reactivity: human, mouse, rat
-Application: WB, IF
-Cell Rep 10(9): 1626-1638.,2015

Mouse anti-Galectin-3 B2C10, 556904, BD Pharmingen
In manuscript: Supplementary Fig. 1
-http://www.bdbiosciences.com/us/applications/research/intracellular-flow/intracellular-antibodies-and-isotype-controls/anti-human-antibodies/purified-mouse-anti-human-galectin-3-b2c10/p/556904
-Species Reactivity: human
-Application: Intracellular staining (flow cytometry), IHC (antigen retrieval required)
- Acta Neuropathol 134(4): 629-653., 2017

Mouse anti-Galectin-3 B2C10 (provided by FT-Liu)
In manuscript: Fig. 2F, Fig. 6A, D, Supplementary Fig. 3A, Supplementary Fig. 4, Supplementary Fig. 6A, Supplementary Fig. 7, Supplementary Fig. 10.
-Species Reactivity: human, mouse
-Application: WB, IF, Flow cytometry
-Biochemistry 35(19): 6073-6079., 1996

Goat anti-Galectin-3 (provided by FT-Liu)
-In manuscript: Fig. 3A, Fig. 4A, Supplementary Fig. 3B, C, Supplementary Fig. 6E, F
-Species Reactivity: human, mouse
-Application: WB, IF, Flow cytometry
-Am J Pathol 147(4): 1016-1028., 1995

Rabbit anti-Galectin-3 (provided by FT-Liu)
-In manuscript: Fig. 2H, Supplementary Fig. 9
-Species Reactivity: human, mouse
-Application: WB, IF, Flow cytometry
-Am J Pathol 147(4): 1016-1028., 1995

NFκB-p65, MAB3026, Millipore
-In manuscript: Fig. 3A, Fig. 4A, Fig. 7B, Supplementary Fig. 11, Supplementary Fig. 13A
-https://www.merckmillipore.com/TW/zh/product/Anti-NFB-Antibody-p65-subunit-active-subunit-clone-12H11,MM_NF-MAB3026?ReferrerURL=https%3A%2F%2Fwww.google.com%2F&bd=1
-Species Reactivity: human, mouse, rat, rabbit
-Application: EMSA, Flow cytometry, ICC, IF, IHC, IH(P), WB
-PLoS One 9(6): e100546., 2014

NFκB-p65, [112A1021] ab13594, Abcam
-In manuscript: Supplementary Fig. 5
-https://www.abcam.com/nf-kb-p65-antibody-112a1021-ab13594.html
-Species Reactivity: mouse, rat, human
-Application: WB, IHC-P, Flow cytometry
-Med Sci Monit 22: 2035-2042., 2016

Phospho-NFκB-p65 (SER536)(93H1), #3033 (Cell Signaling)
-In manuscript: Supplementary Fig. 5
-https://www.cellsignal.com/products/primary-antibodies/phospho-nf-kb-p65-ser536-93h1-rabbit-mab/3033
-Species Reactivity: human, mouse, rat, hamster, monkey, pig
-Application: WB, IP, IF, Flow cytometry
-Sci Rep 9(1): 3825., 2019

LaminB1, GTX103292, GeneTex
-In manuscript: Supplementary Fig. 5
-https://www.genetex.com/Product/Detail/Lamin-B1-antibody/GTX103292
-Species Reactivity: human, mouse, rat, Xenopus laevis, chicken, Rhesus monkey, chimpanzee, bovine
-Application: IHC-P, IP, WB
-Nat Commun 5: 5220., 2014

Tubulin, CP06, Calbiochem
-In manuscript: Supplementary Fig. 5, Supplementary Fig. 10
http://www.merckmillipore.com/TW/zh/product/Anti-Tubulin-Mouse-mAb-DM1A,EMD_BIO-CP06?ReferrerURL=https%3A%2F%2Fwww.google.com%2F&bd=1
-Species Reactivity: human, mouse, rat, chicken
-Application: IF, WB
-Cancer Cell 15(4): 255-269., 2009

Actin, A2066, Sigma
-In manuscript: Fig. 2C
https://www.sigmaaldrich.com/catalog/product/sigma/a2066?lang=en&region=TW
-Species Reactivity: amoeba, chicken, vertebrates, slime mold, human
-Application: IHC, IF, WB
-J Biol Chem 282(8): 5641-5652., 2007

LAMP1, ab24170, Abcam
-In manuscript: Fig. 6A, B, Supplementary Fig. 10
-https://www.abcam.com/lamp1-antibody-lysosome-marker-ab24170.html
-Species Reactivity: mouse, rat, chicken, hamster, cat, dog, human, Xenopus laevis, zebrafish, African green monkey
-Application: IP, IHC-P, WB, IHC-Fr
-EMBO J 38(2)., 2019

LAMP2, ab25339, Abcam
-In manuscript: Supplementary Fig. 7A, Supplementary Fig. 9
-https://www.abcam.com/lamp2-antibody-abl-93-ab25339.html
-Species Reactivity: mouse
-Application: Flow Cytometry, IHC-Fr, IP, ICC/IF, WB
-Nat Commun 9(1): 1808., 2018

LC3, GTX127375, GeneTex
-In manuscript: Supplementary Fig. 7B
-https://www.genetex.com/Product/Detail/LC3B-antibody/GTX127375
-Species Reactivity: human, mouse, pig, rat
-Application: WB, ICC/IF, IHC-P, FACS, IP
-Sci Rep 9(1): 2236., 2019

SDHB,GTX113833, GeneTex
-In manuscript: Supplementary Fig. 7C
-https://www.genetex.com/Product/Detail/SDHB-antibody/GTX113833
-Species Reactivity: human, mouse, rat
-Application: WB, ICC/IF, IHC-P
-Sci Rep 9(1): 2236., 2019

EM48, MAB5374, Millipore
-In manuscript: Fig. 9D
-http://www.merckmillipore.com/TW/zh/product/Anti-Huntingtin-Protein-Antibody-clone-mEM48,MM_NF-MAB5374?ReferrerURL=https%3A%2F%2Fwww.google.com%2F
-Species Reactivity: human, rat, mouse
-Application: ICC, IHC, WB
-Nat Neurosci 17(6): 822-831., 2014

NeuN, ABN78, Millipore
-In manuscript: Fig. 9D, Supplementary Fig. 2B, D, Supplementary Fig. 3C
-http://www.merckmillipore.com/TW/zh/product/Anti-NeuN-Antibody-rabbit,MM_NF-ABN78?ReferrerURL=https%3A%2F%

2Fwww.google.com%2F
-Species Reactivity: human, mouse, rat
-Application: ICC, IHC, IF, WB
-Nat Commun 6: 6807., 2015

NeuN, MAB377, Millipore
-In manuscript: Fig. 9G
-http://www.merckmillipore.com/TW/zh/product/Anti-NeuN-Antibody-clone-A60,MM_NF-MAB377?ReferrerURL=https%3A%2F%2Fwww.google.com%2F
-Species Reactivity: Avian, chicken, ferret, human, mouse, porcine, rat, primate, salamander
-Application: FC, ICC, IF, IHC, IH(P), IP, WB
-Sci Rep 5: 14624., 2015

S100, Z0311, Dako
-In manuscript: Supplementary Fig. 2A, 2C
-https://www.labome.com/product/Dako/Z0311.html
-Species Reactivity: human, mouse, rat, zebrafish
-Application: ICC, IHC, IF, WB
-Glia 65(1): 50-61., 2017

GFAP, G9269, Sigma
-In manuscript: Supplementary Fig. 6E
-https://www.sigmaaldrich.com/catalog/product/sigma/g9269?lang=en&region=TW
-Species Reactivity: human, mouse, rat
-Application: IHC, IF, WB
-Front Neurol 4: 28., 2013

CD68, ab31630, Abcam
-In manuscript: Fig. 7A
-https://www.abcam.com/cd68-antibody-ed1-ab31630.html
-Species Reactivity: mouse, rat, human
-Application: Flow Cytometry, IHC-Fr, IP, WB, RIA, IHC-FrFl, IHC-P
-Nat Commun 9(1): 551., 2018

NLRP3, AG-20B-0014, AdipoGen
-In manuscript: Fig. 8A, Supplementary Fig. 13B
-https://adipogen.com/ag-20b-0014-anti-nlrp3-nalp3-mab-cryo-2.html/
-Species Reactivity: human, mouse
-Application: ICC, IHC, IF, WB, ChIP assay
-Nature 493(7434): 674-678., 2013

DARPP32, #2302, Cell Signaling
-In manuscript: Fig. 9G
-https://www.cellsignal.com/products/primary-antibodies/darpp-32-antibody/2302
-Species Reactivity: human, mouse, rat
-Application: IF, WB
-Sci Rep 8(1): 10068., 2018

CD11b-FITC, 101205, BioLegend
-In manuscript: Fig. 2H
https://www.biolegend.com/en-us/products/fitc-anti-mouse-human-cd11b-antibody-347
-Species Reactivity: mouse, human
-Application: Flow cytometry
-Proc Natl Acad Sci U S A 111(11): E998-1006., 2014

Animals and other organisms

Policy information about studies involving animals; ARRIVE guidelines recommended for reporting animal research

Laboratory animals	Species : mouse<br>Strain : B6CBA-Tg(HDexon1)62Gpb/1J<br>Sex : female<br>Age : 5, 7, 12 weeks old<br><br>Species : mouse<br>Strain : B6.129P2-Htttm2Detl/150J<br>Sex : male and female<br>Age : 15 and 21 months old
Wild animals	The study did not involve wild animals.
Field-collected samples	The study did not involve samples collected from the field.

Ethics oversight	All animal procedures were performed in accordance with the protocols of the Academia Sinica Institutional Animal Care and Utilization Committee, Taiwan.

Note that full information on the approval of the study protocol must also be provided in the manuscript.

Human research participants

Policy information about studies involving human research participants

Population characteristics	The age, gender, genotypic information and diagnosis information were listed as below in the form of mean ± standard deviation:<br>Age : Non-Huntington's disease (Non-HD) individuals, 51.2 ± 12.5; HD individuals, 50.6 ± 12.6<br>Gender : Non-HD individuals, 7 men and 9 women; HD individuals, 14 men and 16 women<br>HD individuals CAG repeats : 44 ± 3<br>Onset age (years) : 45.1 ± 12.7<br>Disease duration (years) : 8.0 ± 8.7
Recruitment	Random age-matched non-HD individuals to HD patients were recruited into study groups.
Ethics oversight	Patients were recruited in the cohort study with approval from the Institutional Review Board and the Ethics Committee of Chang Gung Memorial Hospital, Taipei Veterans General Hospital and Academia Sinica, Taiwan.

Note that full information on the approval of the study protocol must also be provided in the manuscript.

Flow Cytometry

Plots

Confirm that:

- [x] The axis labels state the marker and fluorochrome used (e.g. CD4-FITC).
- [x] The axis scales are clearly visible. Include numbers along axes only for bottom left plot of group (a 'group' is an analysis of identical markers).
- [ ] All plots are contour plots with outliers or pseudocolor plots.
- [x] A numerical value for number of cells or percentage (with statistics) is provided.

Methodology

Sample preparation	The biological sources of the cells were primary cultures of microglia isolated from R6/2 mice and the control mice as described in the Method section. Cells were fixed with BD Cytofix/Cytoperm solution (554722, BD Biosciences) for 20 min at 4 °C. After that, samples were washed and permeabilized twice with BD Perm/Wash buffer (554723, BD Biosciences) before sequential labeling with a rabbit anti-Gal3 antibody, a goat anti-rabbit secondary antibody (Alexa Fluor 568) and a FITC-conjugated CD11b (101205, BioLegend) at 4°C in the dark for 30 min. The samples were washed three times after each labeling and were resuspended in staining buffer before being subjected to flow cytometer.
Instrument	Attune® NxT acoustic focusing flow cytometer (Life Technologies, USA)
Software	Attune NxT Software were used to collect and analyze the flow cytometry data. No custom code has been deposited into a community repository.
Cell population abundance	The purity of the samples (primary microglia) were determined by the labeling of CD11b, a marker that is specific to microglia in the brain tissues. The purity of the cells of WT and R6/2 microglia were 99.70 ± 0.03% and 99.50 ± 0.05%, respectively. Analysis were performed on CD11b and Gal3 double positive fraction, which were 98.08 ± 0.53% and 98.62 ± 0.26% for WT and R6/2 microglia, respectively.
Gating strategy	In these samples gating, samples were first gated to distinguish populations of cells based on their side and forward scatter properties (SSC-A vs FSC-A), followed by singlets (FSC-H vs. FSC-A). To define microglia population, the samples were gated on CD11b-FITC-positive cells. For Gal3 expression analysis, the cells were analyzed from CD11b-FITC and Gal3-568 double positive population (Gal3-Alexa 568-A vs CD11b-FITC-A).

- [x] Tick this box to confirm that a figure exemplifying the gating strategy is provided in the Supplementary Information.
